# A prospective multicenter cohort study of frailty in younger critically ill patients

**DOI:** 10.1186/s13054-016-1338-x

**Published:** 2016-06-06

**Authors:** M. Bagshaw, Sumit R. Majumdar, Darryl B. Rolfson, Quazi Ibrahim, Robert C. McDermid, H. Tom Stelfox

**Affiliations:** Division of Critical Care Medicine (University of Alberta Hospital), Faculty of Medicine and Dentistry, University of Alberta, 2-124 Clinical Sciences Building, 8440-112 Street NW, Edmonton, AB T6G 2B7 Canada; Department of Medicine, Faculty of Medicine and Dentistry, University of Alberta, 5-112 Clinical Sciences Building, 8440-112 Street, Edmonton, AB T6G 2B7 Canada; Division of Geriatric Medicine, Department of Medicine, Faculty of Medicine and Dentistry, University of Alberta, 13-103 Clinical Sciences Building, 8440-112 Street, Edmonton, AB T6G 2B7 Canada; Population Health Research Institute, McMaster University, 237 Barton Street East, Hamilton, ON L8L 2X2 Canada; Department of Medicine, Faculty of Medicine and Dentistry, University of British Columbia, 317-2194 Health Sciences Mall, Vancouver, BC V6T 1Z3 Canada; Department of Critical Care Medicine, Faculty of Medicine, University of Calgary, 2500 University Drive NW, Calgary, AB T2N 1N4 Canada

**Keywords:** Frailty, Critical illness, Intensive care unit, Mortality, Quality of life, Health services

## Abstract

**Background:**

Frailty is a multidimensional syndrome characterized by loss of physiologic and cognitive reserve that heightens vulnerability. Frailty has been well described among elderly patients (i.e., 65 years of age or older), but few studies have evaluated frailty in nonelderly patients with critical illness. We aimed to describe the prevalence, correlates, and outcomes associated with frailty among younger critically ill patients.

**Methods:**

We conducted a prospective cohort study of 197 consecutive critically ill patients aged 50–64.9 years admitted to intensive care units (ICUs) at six hospitals across Alberta, Canada. Frailty was defined as a score ≥5 on the Clinical Frailty Scale before hospitalization. Multivariable analyses were used to evaluate factors independently associated with frailty before ICU admission and the independent association between frailty and outcome.

**Results:**

In the 197 patients in the study, mean (SD) age was 58.5 (4.1) years, 37 % were female, 73 % had three or more comorbid illnesses, and 28 % (*n* = 55; 95 % CI 22–35) were frail. Factors independently associated with frailty included not being completely independent (adjusted OR [aOR] 4.4, 95 % CI 1.8–11.1), connective tissue disease (aOR 6.0, 95 % CI 2.1–17.0), and hospitalization within the preceding year (aOR 3.3, 95 % CI 1.3–8.1). There were no significant differences between frail and nonfrail patients in reason for admission, Acute Physiology and Chronic Health Evaluation II score, preference for life support, or treatment intensity. Younger frail patients did not have significantly longer (median [interquartile range]) hospital stay (26 [9–68] days vs. 19 [10–43] days; *p* = 0.4), but they had greater 1-year rehospitalization rates (61 % vs. 40 %; *p* = 0.02) and higher 1-year mortality (33 % vs. 20 %; adjusted HR 1.8, 95 % CI 1.0–3.3; *p* = 0.039).

**Conclusions:**

Prehospital frailty is common among younger critically ill patients, and in this study it was associated with higher rates of mortality at 1 year and with rehospitalization. Our data suggest that frailty should be considered in younger adults admitted to the ICU, not just in the elderly. Additional research is needed to further characterize frailty in younger critically ill patients, along with the ideal instruments for identification.

**Electronic supplementary material:**

The online version of this article (doi:10.1186/s13054-016-1338-x) contains supplementary material, which is available to authorized users.

## Background

Frailty is a multidimensional syndrome characterized by a decline in physiologic and cognitive homeostatic reserve that increases susceptibility to adverse events and unfavorable outcomes, often following relatively minor stressors [1]. Frailty is causally related to aging, has been conventionally described in elderly populations, and characterizes a common trajectory at the end of life [2–5]. Frail persons show greater risk for procedural complications, disability, impaired health-related quality of life (HRQoL), hospitalizations, institutionalization, and death [6–10].

Recent data show that frailty is common among patients admitted to intensive care units (ICUs) [11, 12]. Premorbid frailty appears to be an independent (and potentially modifiable) factor associated with less favorable outcomes and greater health services use [13–15]. However, most studies have been focused exclusively on describing frailty among older populations (i.e., ≥65 years of age); in fact, frailty has rarely been considered to occur in the nonelderly [12, 14–16]. The prevalence of frailty in the Canadian general population among persons aged 40–69 years is estimated at <10 %; however, when present, it portends greater health service use and mortality risk [17].

We hypothesized frail patients may have greater susceptibility to developing critical illness and that their risk may be “age-shifted” compared with nonfrail patients of similar chronologic age. Consequently, the prevalence of frailty may be higher than expected for chronologic age among a cohort of younger patients admitted to the ICU [17]. Previously, we performed a prospective multicenter study in a cohort of critically ill patients to describe the association between frailty and outcomes [11]. In this substudy, we aimed to specifically examine the prevalence, correlates, and outcomes associated with frailty in a younger cohort of critically ill patients (i.e., age at ICU admission 50–64.9 years), in whom it has rarely been described.

## Methods

### Study design, participants and setting

We conducted a planned substudy of a prospective multicenter cohort study that has been described previously [11]. Adults admitted to six closed multisystem medical-surgical ICUs located in two tertiary/academic and four community hospitals in Alberta, Canada, between February 2010 and July 2011 were screened for enrollment [11]. The study was approved by the research ethics board at the University of Alberta (Pro00007628). All participants or their designated surrogate decision-makers provided written informed consent to participate.

### Frailty definition

Frailty was operationalized using the Canadian Study of Health and Aging Clinical Frailty Scale (CFS) score, which was modified to an 8-point tool designed to categorize patients as fit, vulnerable, or frail [6]. The CFS is a subjective judgment-based screening tool for frailty that has been proven to be valid, reliable, and simple to perform. We defined patients as frail if their CFS score was ≥5 (moderate to severe frailty for CFS score 6–8), as vulnerable if their CFS score was 4, and as fit if their CFS score was ≤3 [11]. Trained coordinators interviewed participants and/or their surrogate decision-makers and reviewed each participant’s medical record.

### Outcomes

The primary outcome was all-cause mortality 1 year following enrollment. Secondary outcomes were focused on (1) patient-centered outcomes, including ICU, hospital, 90-day, and 6-month mortality; HRQoL at 6 and at 12 months, captured using the EuroQol (EQ-5D) Health Questionnaire (including the EQ-5D visual analogue scale [EQ-5D-VAS]) [13]; and discharge disposition; and (2) health service use, including ICU and hospital lengths of stay, ICU readmission, and hospital readmission in the 1-year period following enrollment.

### Data collection and management

Data were prospectively captured on standardized forms and entered into an electronic database. These data elements included sociodemographic factors, baseline functional status and disability (e.g., basic and instrumental activities of daily living), comorbid conditions defined and summated using the Elixhauser comorbidity scale [18], prescription medications, source of ICU admission (e.g., ward, operating theater, emergency department), diagnostic category, illness severity (e.g., defined according to the Acute Physiology and Chronic Health Evaluation [APACHE] II score [19]), presence and severity of organ dysfunction (e.g., defined according to the Sequential Organ Failure Assessment score [20]), treatment intensity (e.g., mechanical ventilation, vasoactive support, renal replacement therapy), and preferences for life-sustaining therapy (e.g., full ICU support, limitation in therapy). Participants were contacted at 6 and 12 months to ascertain long-term outcomes, including vital status, disposition, and HRQoL.

### Statistical analyses

Among nonelderly patients, the distribution of CFS scores was presented and descriptive statistics were calculated according to the presence or absence of frailty. Independent associations between baseline sociodemographic and clinical characteristics as well as frailty status were evaluated using multivariable logistic regression analysis. Clinically important variables (i.e., sex, comorbidity, case mix, APACHE II score, site) and those found to be significant in univariate analysis (*p* = 0.20) were entered into the multivariable model. Model calibration and discrimination were assessed using the Hosmer-Lemeshow goodness-of-fit test, Brier score, and the AUC (c-statistic). The independent percentage contribution of each variable in the model’s explanatory power was estimated by dividing differences in log-likelihoods of the nested models by the difference in the log-likelihoods of null and full (final) models [14, 21]. Sensitivity analysis was performed by adding the variables age, surgical status, and sepsis to the multivariable model. Survival curves were plotted using Kaplan-Meier curves with log-rank tests. Multivariable Cox proportional hazards regression analyses were performed to analyze survival. Similar to the analyses described above, clinically important variables (i.e., sex, comorbidity, case mix, APACHE II score, site) and those found to be significant in univariate analysis (*p* = 0.20) were entered into the multivariable model. Proportional hazards model assumptions were checked by comparing log (−log) plots of survival probabilities over time of frail and nonfrail patients and testing interactions between frailty status and logarithmic scales of follow-up times in the model. A *p* value <0.05 was considered statistically significant for all comparisons. All analyses were performed using STATA 12.1 software (StataCorp, College Station, TX, USA).

## Results

**Fig. 1:**
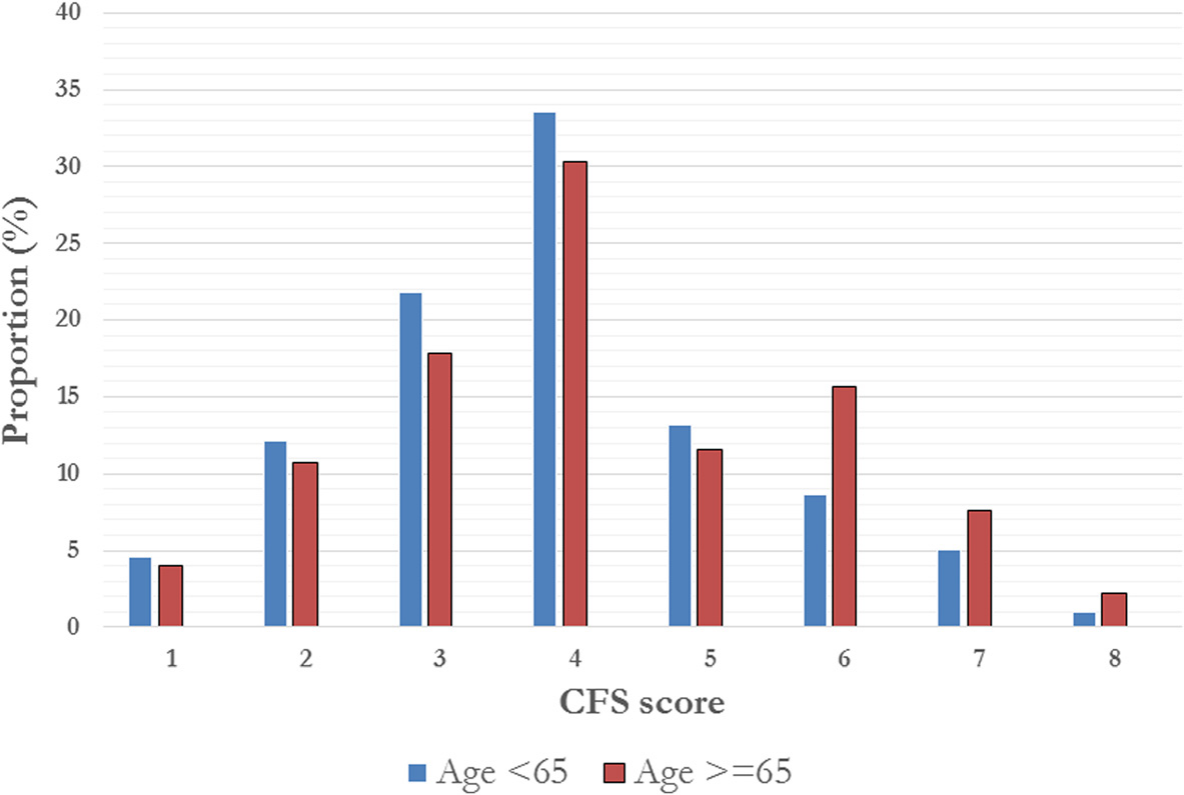
Summary of prevalence of Clinical Frailty Scale (CFS) scores, stratified by age older or younger than 65 years

Overall, 197 patients (47 % of the study cohort) aged 50–64.9 years were included in this substudy (Additional files 1 and 2). The mean (SD) age was 58.5 (4.1) years, 37 % (*n* = 72) were female, 73 % (*n* = 143) had three or more comorbid illnesses, 74 % (*n* = 146) were living at home independently, and 41 % (*n* = 80) had been hospitalized in the preceding 1-year period. The median (interquartile range) prehospital CFS score was 4 (3–5). Of the cohort, 39 % (95 % CI 32–46; *n* = 76) were classified as fit, 34 % (95 % CI 27–41 %; *n* = 66) were classified as vulnerable, and 28 % (95 % CI 22–35 %; *n* = 55) were classified as frail (CFS score ≥5) (Fig. 1).

### Factors associated with frailty among nonelderly patients

There were numerous differences in baseline sociodemographic and clinical characteristics among frail and nonfrail patients (Table 1). Several factors were found in multivariable analysis to be independently associated with prehospital frailty, including prehospital residence, receipt of disability insurance, prior hospitalization, female sex, and comorbid connective tissue disease (CTD) (Table 2). These five variables represented 89 % of the final model’s explanatory power for prehospital frailty.

**Table 1:** Baseline sociodemographic, clinical, and comorbidity data for patients younger than 65 years old admitted to the intensive care unit, stratified by frailty status

Variable	Frail (*n* = 55, 28 %)	Nonfrail (*n* = 142, 72 %)	*p* Value
Age, years, mean ± SD	58.9 ± 4.1	58.4 ± 4.2	0.444
Sex, female, *n* (%)	28 (50.9)	44 (31.0)	0.009
Widowed, *n* (%)	4 (7.3)	7 (4.9)	0.504
Education, *n* (%)			0.039
Less than secondary school	14 (25.5)	17 (12.0)	
Secondary school	23 (41.8)	58 (40.8)	
Higher-level degree	18 (32.7)	67 (47.2)	
Employment status, *n* (%)			
Full-time	9 (16.4)	50 (35.2)	0.010
Part-time	1 (1.8)	15 (10.6)	0.045
On disability	28 (50.9)	31 (21.8)	<0.001
Prehospital residence, *n* (%)			<0.001
At home (independent)	23 (41.8)	123 (86.6)	
At home (with help)	26 (47.3)	18 (12.7)	
Other	6 (10.9)	1 (0.7)	
CSHA Function Scale score (n, %)			
Eating (independent)	51 (92.7)	142 (100)	0.006
Dressing (independent)	47 (85.5)	141 (99.3)	<0.001
Personal care (independent)	45 (81.8)	142 (100)	<0.001
Walking (independent)	35 (63.6)	134 (94.4)	<0.001
Getting out of bed (independent)	40 (72.7)	141 (99.3)	<0.001
Taking bath (independent)	37 (67.3)	140 (98.6)	<0.001
Using toilet (independent)	49 (89.1)	140 (98.6)	0.007
Using telephone (independent)	52 (94.5)	142 (100)	0.021
Going shopping (independent)	25 (45.5)	133 (93.7)	<0.001
Preparing own meals (independent)	29 (52.7)	138 (97.2)	<0.001
Doing housework (independent)	26 (47.3)	132 (93.0)	<0.001
Taking medicine (independent)	40 (72.7)	133 (93.7)	<0.001
Managing own finances (independent)	46 (83.6)	138 (97.2)	0.002
Elixhauser comorbidity score, mean ± SD	8.7 ± 9.1	6.6 ± 7.6	0.098
Hypertension	26 (47.3)	73 (51.4)	0.602
Heart failure	10 (18.2)	9 (6.3)	0.012
Diabetes mellitus	18 (32.7)	31 (21.8)	0.112
Chronic kidney disease	13 (23.6)	23 (16.2)	0.225
Rheumatoid/connective tissue disease	20 (36.4)	13 (9.2)	<0.001
Any cancer	5 (9.1)	16 (11.3)	0.657
Alcohol/drug abuse	16 (29.1)	50 (35.2)	0.414
Psychosis	3 (5.5)	4 (2.8)	0.401
Depression	18 (32.7)	38 (26.8)	0.405
Prescription medications, *n*, mean ± SD	8.8 ± 6.2	5.1 ± 4.3	<0.001
Hospitalization in preceding 1-year period, mean ± SD	34 (61.8)	46 (32.4)	<0.001

*CSHA* Canadian Study on Health and Aging

**Table 2:** Multivariable analysis of factors associated with prehospital frailty among patients younger than 65 years old

Variable	Adjusted OR (95 % CI)	*p* Value	% contribution
Sex			3.8
Male	1		
Female	2.01 (0.87–4.66)	0.103	
Elixhauser comorbidity score, mean ± SD	0.96 (0.91–1.02)	0.213	1.6
Rheumatoid/CTD	6.00 (2.12–17.0)	0.001	19.7
Heart failure	3.28 (0.78–13.7)	0.104	1.8
Prehospital residence			49.3
At home (independent)	1		
At home (with help)/other	4.40 (1.75–11.1)	0.002	
On disability	2.11 (0.83–5.35)	0.117	4.1
Managing own finances (independent)	0.24 (0.05–1.24)	0.088	3.5
Never married	0.23 (0.03–1.56)	0.133	2.8
Education			1.7
Less than high school	1		
High school	0.76 (0.23–2.50)	0.653	
Higher-level degree	0.53 (0.16–1.75)	0.296	
Prescription medications, *n*, mean ± SD	1.01 (0.93–1.10)	0.748	0.1
Prior hospitalization	3.29 (1.34–8.10)	0.010	11.7

*CTD* connective tissue disease

The overall model is significant (likelihood ratio χ_12_^2^ = 78.73 with *p* < 0.0001) with good discriminatory ability (c-statistic 0.85) and goodness of fit (calibration, Brier score 0.12; Hosmer-Lemeshow χ_8_^2^ = 10.37 with *p* = 0.24). In sensitivity analyses, age, surgical status, and sepsis were also included in the multivariable model. These did not translate into significant changes across any covariates or percentage contribution to the model.

### Association between frailty and clinical course among nonelderly patients

Other than postoperative status (which was less common among frail patients), there were no significant differences in diagnostic category, admission source, treatment intensity or patient preferences for life-sustaining therapy between frail and not frail (Table 3).

**Table 3:** Summary of case mix, clinical course, and outcomes for critically ill patients younger than 65 years old, stratified by frailty status

Variable	Frail	Nonfrail	*p* Value
ICU diagnostic category, *n* (%)			0.284
Sepsis	10 (18.2)	15 (10.6)	
Cardiovascular	5 (9.1)	20 (14.1)	
Respiratory	22 (40.0)	45 (31.7)	
Gastrointestinal/liver	9 (16.4)	25 (17.6)	
Other^a^	9 (16.4)	37 (26.1)	
ICU admission source, *n* (%)			0.900
Ward transfer	17 (30.9)	41 (28.9)	
OR theater transfer	13 (23.6)	39 (27.5)	
ED	14 (25.5)	40 (28.1)	
Other^b^	10 (18.2), 1 (1.8)	20 (14.1), 2 (1.4)	
Postoperative, *n* (%)	13 (23.6)	55 (38.7)	0.046
APACHE II score, mean ± SD	19.8 ± 6.7	17.9 ± 7.4	0.103
SOFA score, mean ± SD	8.2 ± 4.0	7.0 ± 4.1	0.086
Mechanical ventilation, *n* (%)	52 (94.5)	120 (84.5)	0.058
Vasoactive medications, *n* (%)	33 (60.0)	72 (50.7)	0.241
Renal replacement therapy, *n* (%)	6 (10.9)	21 (14.8)	0.478
Preferences for support, *n* (%)			0.069
Full ICU support	48 (87.3)	134 (95.0)	
Limitations on therapy (DNR order)	7 (12.7)	7 (5.0)	
Mortality, *n* (%)			
ICU	6 (10.9)	10 (7.0)	0.390
Hospital	11 (20.0)	20 (14.1)	0.306
90-day	13 (23.6)	21 (14.8)	0.140
6-month	16 (29.1)	25 (17.6)	0.075
1-year	18 (32.7)	29 (20.4)	0.069
ICU length of stay, days, median (IQR)	6 (3.5–11.5)	6 (3–10)	0.383
ICU readmission, *n* (%)	9 (18.4)	18 (13.6)	0.427
Hospital length of stay, median (IQR)	26 (9–68)	18.5 (10–43)	0.389
Hospital readmission, *n* (%)	26 (60.5)	49 (40.2)	0.022
Discharge disposition, *n* (%)			0.112
Living at home independent	14 (32.6)	62 (50.8)	
Living at home with help	16 (37.2)	31 (25.4)	
Other	13 (30.2)	29 (23.8)	
EQ-5D-VAS, 6-month			
Mean (SD)	58.8 (18.7)	63.4 (20.4)	0.254
*n* (%)	34/39 (87.2)	96/117 (82.1)	
EQ-5D-VAS, 1-year			
Mean (SD)	63.0 (20.2)	68.3 (17.6)	0.184
*n* (%)	28/37 (75.7)	85/113 (75.2)	
MCID^c^ in EQ-5D-VAS between 6 and 12 months, *n* (%)	10/27 (37.0)	29/77 (37.7)	0.95
EQ-5D, 6-month, *n* (%)			
Mobility	25 (71.4)	40 (41.2)	0.002
Self-care	11 (31.4)	8 (8.2)	0.001
Usual activities	30 (85.7)	65 (67.7)	0.041
Pain/discomfort	30 (85.7)	54 (55.7)	0.002
Anxiety/depression	19 (54.3)	36 (37.1)	0.077

*ICU* intensive care unit, *ED* emergency department, *OR* operating room, *VAS* visual analogue scale, *DNR* do not resuscitate, *APACHE* Acute Physiology and Chronic Health Evaluation, *SOFA* Sequential Organ Failure Assessment, *EQ-5D* EuroQol Health Questionnaire, *MCID* minimal clinically important difference, *IQR* interquartile range

^a^Other was defined as urologic/renal, neurologic, endocrinologic/metabolic, hematologic/oncologic, trauma, musculoskeletal

^b^Other was defined as transfer from another hospital, other location

^c^Minimum difference of 7 points in the EQ-5D-VAS was considered clinically important [13]

### Association between frailty and mortality among nonelderly patients

**Fig. 2:**
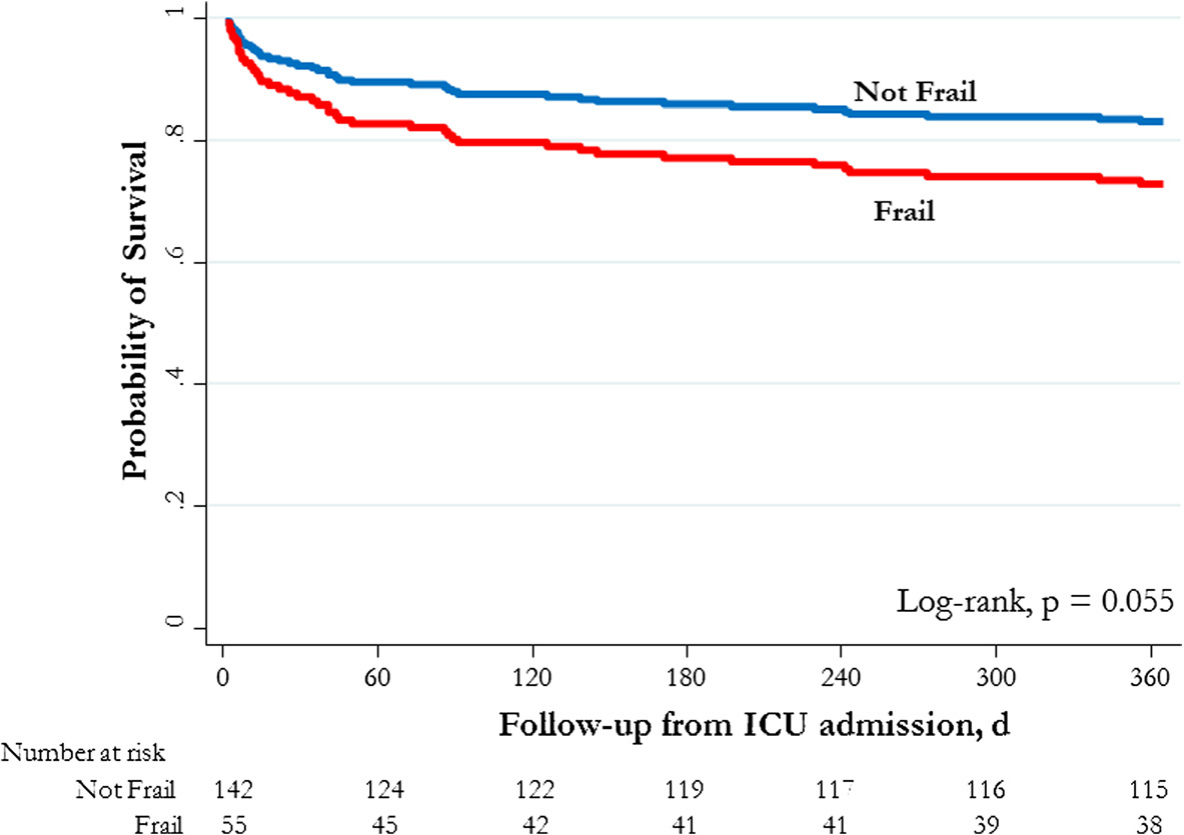
Adjusted survival probabilities by frailty status in intensive care unit (ICU) patients younger than 65 years old. Survival curves adjusted for sex, Elixhauser comorbidity score, Acute Physiology and Chronic Health Evaluation II score, primary diagnostic criteria, and hospital type (tertiary care/academic vs. community hospital)

**Fig. 3:**
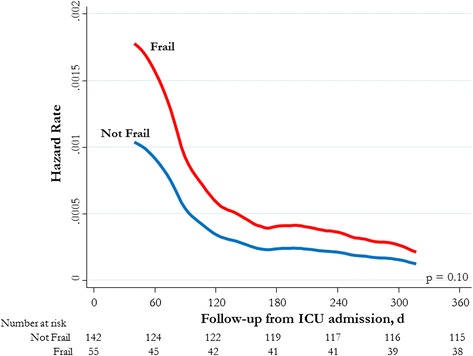
Adjusted hazard rates of death stratified by frailty status in intensive care unit (ICU) patients younger than 65 years old. Survival curves adjusted for sex, Elixhauser comorbidity score, Acute Physiology and Chronic Health Evaluation II score, primary diagnostic criteria, and hospital type (tertiary care/academic v. community hospital)

**Fig. 4:**
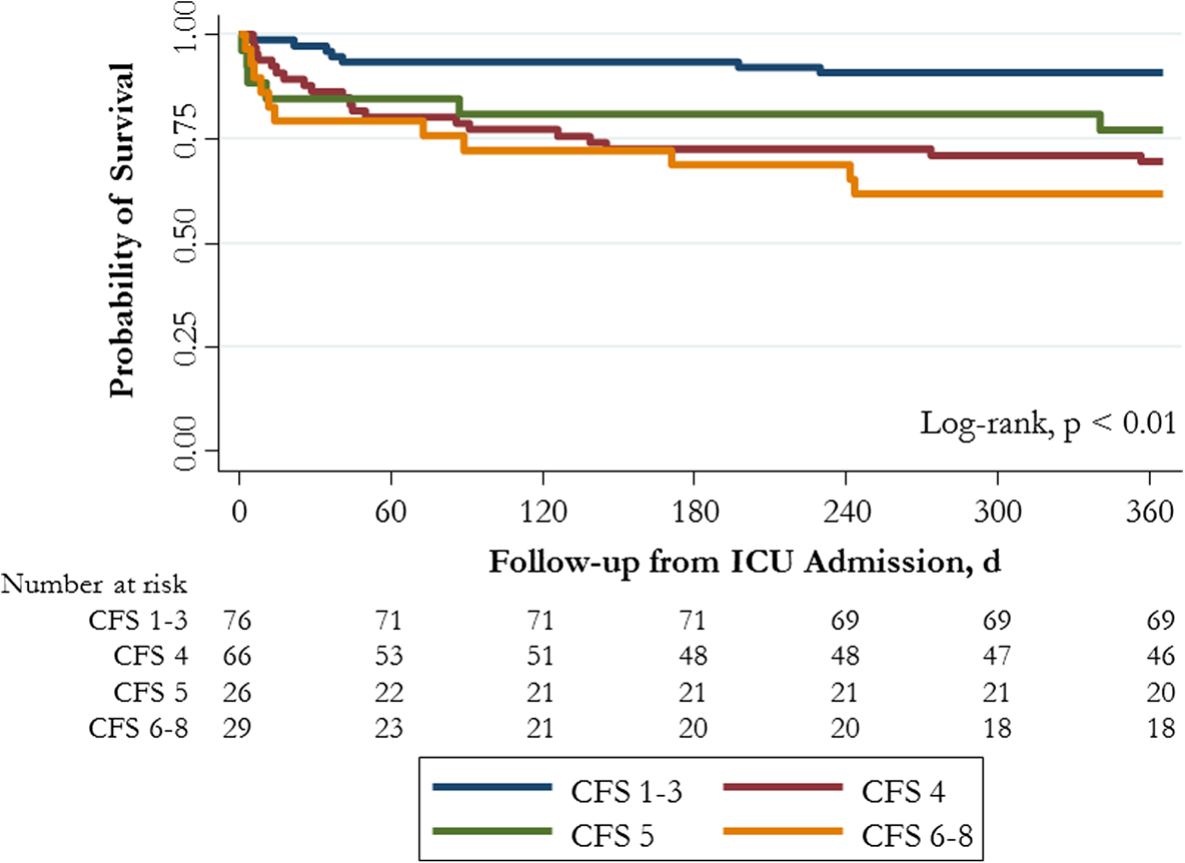
Kaplan-Meier survival curves stratified by Clinical Frailty Scale (CFS) score categories (fit, vulnerable, frail, moderate to severe frailty) in intensive care unit (ICU) patients younger than 65 years old

Unadjusted mortality at 1 year was not significantly greater for frail patients than for nonfrail patients (32.7 % vs. 20.4 %; OR 1.90, 95 % CI 0.95–3.78). Unadjusted mortality in the ICU, in the hospital, at 90 days, and at 6 months was similarly not statistically different between frail and nonfrail patients (Table 3). In multivariable analysis, frail patients were found to have a higher adjusted risk of death at 1 year than nonfrail patients (adjusted HR 1.83, 95 % CI 1.03–3.25; *p* = 0.039) (Fig. 2 and Fig. 3). The burden of comorbid illness, acute illness severity, and frailty represented 81 % of the model’s final explanatory power for 1-year mortality. There was evidence of a dose-response increase in adjusted risk of death associated with greater CFS score (Table 4 and Fig. 4). This increase was most apparent when we compared fit (CFS score 1–3) with moderate to severe frailty (CFS score 6–8), while there was overlap among those classified as vulnerable (CFS score 4) and mildly frail (CFS score 5). In an exploratory analysis using the entire study cohort (*n* = 421), there was an effect modification between frailty and age with respect to all-cause mortality (mortality among frail patients age <65 years 36 % vs. age ≥65 years 59 %; OR 2.0,3 95 % CI 1.30–3.16; *p* < 0.001), although in multivariable analysis the interaction term was not statistically significant (OR 0.84, 95 % CI 0.42–1.71; *p* = 0.68) (Additional file 3).

**Table 4:** Crude and adjusted HR for death by Clinical Frailty Scale score categories in patients younger than 65 years old

CFS category	Unadjusted HR	95 % CI	*p* Value	Adjusted HR^a^	95 % CI	*p* Value
Fit (CFS score 1-3)	1.0	–	–	1.0	–	–
Vulnerable (CFS score 4)	3.67	1.55–8.69	0.003	2.89	1.19–7.02	0.019
Mild frailty (CFS score 5)	2.82	0.95–8.38	0.063	2.54	0.82–7.90	0.107
Moderate to severe frailty (CFS score 6–8)	4.93	1.91–12.73	0.001	4.41	1.62–12.06	0.004

*CFS* Clinical Frailty Scale

^a^Adjusted by sex, Elixhauser comorbidity score, Acute Physiology and Chronic Health Evaluation II score, primary diagnostic criteria, and hospital type (tertiary care/academic vs. community hospital)

### Association between frailty and nonfatal outcomes among nonelderly patients

Among hospital survivors, 69.8 % of frail and 76.2 % of nonfrail patients were living at home (difference 6.4 %, 95 % CI −9.9 % to 22.1 %); however, only 32.8 % of frail patients were independent (absolute decrease from prehospital level −9.2 %, 95 % CI −28.4 % to 9.9 %), while 50.8 % of nonfrail patients were independent (absolute decrease from prehospital −35.8 %, 95 % CI −46.3 % to −25.3 %) at the time of hospital discharge. EQ-5D-VAS scores were similar for frail and nonfrail patients at 6 months (58.8 [18.7] vs. 63.4 [20.4]; *p* = 0.25) and at 12 months (63.0 [20.2] vs. 68.3 [17.6]; *p* = 0.18). There was no significant difference between 6 and 12 months in the EQ-5D-VAS for frail and nonfrail patients (4.0 [95 % CI −1.4 to 9.5] vs. 5.3 [95 % CI 1.8–8.8]; *p* = 0.72) or in the proportion achieving a minimal clinically important improvement in EQ-5D-VAS (37.0 % vs. 37.7 %; *p* = 0.95) by 12 months. A greater proportion of frail patients had problems across all EQ-5D domains compared with those who were not frail (Table 3).

### Association between frailty and health services use among nonelderly patients

There were no statistical differences in ICU or hospital lengths of stay or rates of ICU readmission between frail and nonfrail patients (Table 3). The hospital readmission rate in the year following enrollment was significantly greater for frail patients than for nonfrail patients.

## Discussion

We performed a planned subgroup study to describe the prevalence, as well as to characterize the correlates and outcomes associated with frailty among a younger cohort of critically ill patients. Frailty has customarily been described only among older persons; however, we believe our study provides new knowledge and novel insights into the occurrence and impact of frailty among younger critically ill patients.

### Main findings

First, we showed that frailty was relatively common among younger patients admitted to the ICU in our study, evident in more than one in four. This was significantly greater than the estimated prevalence of frailty among a contemporaneous general population [17]. Second, as expected, several sociodemographic factors correlated with prehospital frailty. Frailty was more common among women, those with less than a secondary school education, those receiving disability insurance, and those requiring assistance at home. In addition, in the 1-year period before the study, health service use was higher among those classified as frail than among those who were not frail. Third, frail patients in this younger cohort where characterized by a high burden of comorbid disease, in particular CTD and heart failure (HF), and were prescribed a greater number of medications than those who were not frail. Fourth, preferences for life support, reasons for ICU admission, and treatment intensity were similar between frail and nonfrail patients. Finally, in adjusted analyses, long-term mortality and rehospitalization were greater among those with prehospital frailty.

### Context with prior literature

Most published literature is focused on describing frailty among older persons [12, 14, 15]; few authors have characterized its epidemiology among nonelderly persons [17], in particular in the context of critical illness [22]. This is likely attributable to most frailty research being performed in the domain of geriatric medicine [6, 16] and using administrative databases with age thresholds (e.g., U.S. Medicare beneficiaries database) [14, 15] or being based on the misconception that frailty is solely a product of chronologic aging [12]. More recently, researchers in a number of studies have evaluated the prevalence and impact of frailty among specialized cohorts of much younger patients, including lung transplant candidates (median age 59 years [23]), end-stage liver disease (mean age 55 years [24]), end-stage kidney disease (mean age 55 years [25]), and kidney transplant (mean age 53 years [26]). A greater rate of deficit accumulation in selected younger persons may accelerate the development of frailty, and, as such, these persons may have “age-shifted” vulnerability to major stressors (i.e., critical illness) [27]. These patients manifest frailty and “age” more rapidly relative to chronologic age [28]. Similarly, the probability of survival after critical illness appears higher for younger than for older frail patients [11]; however, younger patients may still experience a longer and/or more complex post-ICU course and recovery [29–32]. Accruing evidence, along with our data, suggests that selected younger patients, such as those with advanced chronic inflammatory diseases (e.g., CTD) or end-stage organ diseases (e.g., HF, cirrhosis) may benefit from screening for frailty.

Comorbid disease and frailty are not mutually exclusive and may show greater correlation among younger patients with significant and/or advanced chronic illness [1]. As examples, we identified both CTD and HF as predictors of prehospital frailty. Few studies have evaluated the prevalence and impact of frailty among patients with CTD [33]. Numerous factors may predispose these patients to frailty, including chronic disease progression and persistent inflammation, disease-specific therapy (i.e., immunosuppressive or other disease-modifying antirheumatic drugs), nutritional alterations (i.e., cachexia), and sarcopenia [34]. Indeed, sarcopenia and nutritional deficiencies before critical illness may amplify early skeletal muscle loss in critical illness, further prolonging recovery and exacerbating risk of incident or worsening disability [35, 36]. Likewise, an estimated 18–54 % of patients with HF are clinically frail [37]. Frailty in HF may have important prognostic implications, including reduced likelihood of self-management, impaired HRQoL, greater hospitalizations, being declined for transplant, and death [37, 38]. We believe our finding of associations between frailty, selected comorbid conditions, and critical illness requires confirmation and evaluation in larger studies.

Prehospital frailty portends a greater risk of death and impaired recovery following the stress of critical illness. This finding would appear robust regardless of age; however, there is likely some additive effect between frailty, older age, and risk of death [11, 12, 16]. Our study suggests that the association between frailty and mortality may be attenuated in younger compared with older patients; however, those with moderate to severe frailty still exhibit significantly higher risk [11]. This implies that frailty among younger patients may be less likely a terminal event or imminent end-of-life trajectory. That said, these relatively young frail survivors have greater opportunity and time at risk for the multifarious physical (i.e., disability) and psychosocial (i.e., depression, posttraumatic stress, impaired HRQoL, inability to work, lost income, social isolation) complications increasingly described after critical illness [29–32, 39]. Indeed, at 6 months, frail survivors in our study described far greater problems with mobility, self-care, usual activities, and pain. We found that more than half of frail survivors described issues with depression and/or anxiety [40]. Interestingly, despite all EQ-5D domains being generally worse among frail patients, EQ-5D-VAS scores, while impaired, were not significantly different at 6 and 12 months between frail and nonfrail patients. Thus, frail survivors’ self-rated global HRQoL was not significantly different from that of those who were not frail. There may be a number of explanations for these findings. First, it may imply that frail survivors adapt with time to their new vocation and/or disposition, despite the high prevalence of residual “problems.” Second, this substudy may have had limited capability to detect significant clinical differences in HRQoL due to being relative small and within the context of the operative features of the EQ-5D. Regardless, this also translated into greater rates of rehospitalization in the subsequent 1-year period, implying greater health service use and healthcare costs for those screened as frail [5].

### Implications for clinicians and future research

Our study has relevance for intensivists and all the other clinicians who care for survivors of critical illness. It also reinforces the potential value of frailty screening among selected younger patients admitted to the ICU. Recognition and acknowledgement of prehospital frailty can serve to inform survivorship expectations after critical illness, as well as steer the mobilization of customized recovery needs, both in the hospital and in the community, across physical, emotional, and social domains [41]. The ideal content and implementation of multifactorial, interprofessional post-ICU interventions to improve recovery remain uncertain and a challenge for providers [42, 43]. Importantly, we believe further rigorous research in larger cohorts is needed to confirm our findings, to further characterize those younger patients most likely to benefit from frailty screening, and to develop translatable interventions aimed at enhancing recovery (i.e., preserving autonomy, slowing health status deterioration, societal reintegration and engagement) and informing clinical decision-making [13]. Moreover, future work should ideally evaluate the comparative performance of additional screening instruments in addition to the CFS score (e.g., frailty index, physical performance measures). In addition, one of the most important implications of our work is that future research related to recognizing and mitigating frailty should not necessarily be age-restricted. Indeed, on the basis of the importance of prior hospitalizations and the presence of selected comorbid conditions, we believe that the cohort of younger frail patients is only likely to increase over time.

### Limitations

Our study has important limitations that must be considered. First, while the study was preplanned, we recognize that it entails a secondary analysis focused on a smaller subgroup with limited statistical power. Second, while this study was focused on younger critically ill patients, the age range for this subgroup was still limited to patients aged 50–64.9 years. We therefore cannot comment on the prevalence or implications of frailty among those younger than 50 years old. Third, we recognize that our study is susceptible to selection bias, given that all participants were recruited following ICU admission. Fourth, the CFS score was intended as a screening tool for frailty that was previously validated in older patients. We recognize there is no “gold standard” for the diagnosis of frailty among younger patients; however, we believe that the CFS is simple, has face validity, and was able to discriminate a subgroup at increased risk for adverse outcomes [6, 9]. We also did not capture additional highly correlated surrogates for frailty, such as sarcopenia, that were shown to have similar predictive capacity as measures of frailty for adverse outcomes among critically ill surgical patients [44]. Finally, as previously described [13], we did not capture physical performance measures such as mobility, grip strength, or cognition before critical illness, which may have particular relevance among younger frail survivors of critical illness.

## Conclusions

Frailty is common among younger critically ill patients and predicts higher mortality, rates of rehospitalization at 1 year among those who survive critical illness. Frailty needs to be recognized and integrated into management of selected younger patients admitted to the ICU, and not just the elderly. A better understanding of the implications and outcomes associated with prehospital frailty among younger critically ill patients will inform prognostication; contribute to better-informed decision-making; help to manage the survivorship expectations for both patients and their families; and, importantly, guide innovative research focused on interventions.

## Key messages

- Frailty was common among younger ICU patients, being present in an estimated one-fourth of those aged 50–64.9 years.
- Frail patients aged 50–64.9 years were more likely to be female, to have greater comorbid illness (in particular connective tissue disease), and to have impaired baseline function and disability.
- Frail patients aged 50–64.9 years were more likely to have been hospitalized in the 1-year period preceding ICU admission.
- Frail patients aged 50–64.9 years had higher adjusted mortality at 1 year and greater use of healthcare services.

## Abbreviations

APACHE, Acute Physiology and Chronic Health Evaluation; CFS, Clinical Frailty Scale; CSHA, Canadian Study on Health and Aging; CTD, connective tissue disease; DNR, do not resuscitate; ED, emergency department; EQ-5D, EuroQol Health Questionnaire; HF, heart failure; HRQoL, health-related quality of life; ICU, intensive care unit; IQR, interquartile range; MCID, minimal clinically important difference; SOFA, Sequential Organ Failure Assessment; VAS, visual analogue scale

## Additional files

Additional file 1: Summary of participant flow in the study. (TIF 171 kb) Additional file 2: Comparison of baseline sociodemographic, clinical, and comorbidity data for patients in the full study cohort and the subgroup aged <65 years old admitted to the ICU, stratified by frailty status. (DOCX 22 kb) Additional file 3: Multivariable Cox proportional hazards model HR for death in the complete study cohort (*n* = 421). (DOCX 18 kb)
