# Erratum to: A prospective multicenter cohort study of frailty in younger critically ill patients

**DOI:** 10.1186/s13054-016-1393-3

**Published:** 2016-07-19

**Authors:** Sean M. Bagshaw, Sumit R. Majumdar, Darryl B. Rolfson, Quazi Ibrahim, Robert C. McDermid, H. Tom Stelfox

**Affiliations:** Division of Critical Care Medicine (University of Alberta Hospital), Faculty of Medicine and Dentistry, University of Alberta, 2-124 Clinical Sciences Building, 8440-112 Street NW, Edmonton, AB T6G 2B7 Canada; Department of Medicine, Faculty of Medicine and Dentistry, University of Alberta, 5-112 Clinical Sciences Building, 8440-112 Street, Edmonton, AB T6G 2B7 Canada; Division of Geriatric Medicine, Department of Medicine, Faculty of Medicine and Dentistry, University of Alberta, 13-103 Clinical Sciences Building, 8440-112 Street, Edmonton, AB T6G 2B7 Canada; Population Health Research Institute, McMaster University, 237 Barton Street East, Hamilton, ON L8L 2X2 Canada; Department of Medicine, Faculty of Medicine and Dentistry, University of British Columbia, 317-2194 Health Sciences Mall, Vancouver, BC V6T 1Z3 Canada; Department of Critical Care Medicine, Faculty of Medicine, University of Calgary, 2500 University Drive NW, Calgary, AB T2N 1 N4 Canada

## Erratum

Unfortunately, the original version of this article [1] contained an error. The first name of the first author had been omitted. The authors given name was published as ‘M’ and family name as ‘Bagshaw’, however the given name should be ‘Sean M’ and family name should be ‘Bagshaw’. The name is included correctly here and this will be corrected in the original article also.
