# Supplementary figures and images for: A prospective multicenter cohort study of frailty in younger critically ill patients
Source: Crit Care. 2016 Jun 6;20:175. doi: 10.1186/s13054-016-1338-x (PMC4893838; doi:10.1186/s13054-016-1338-x)

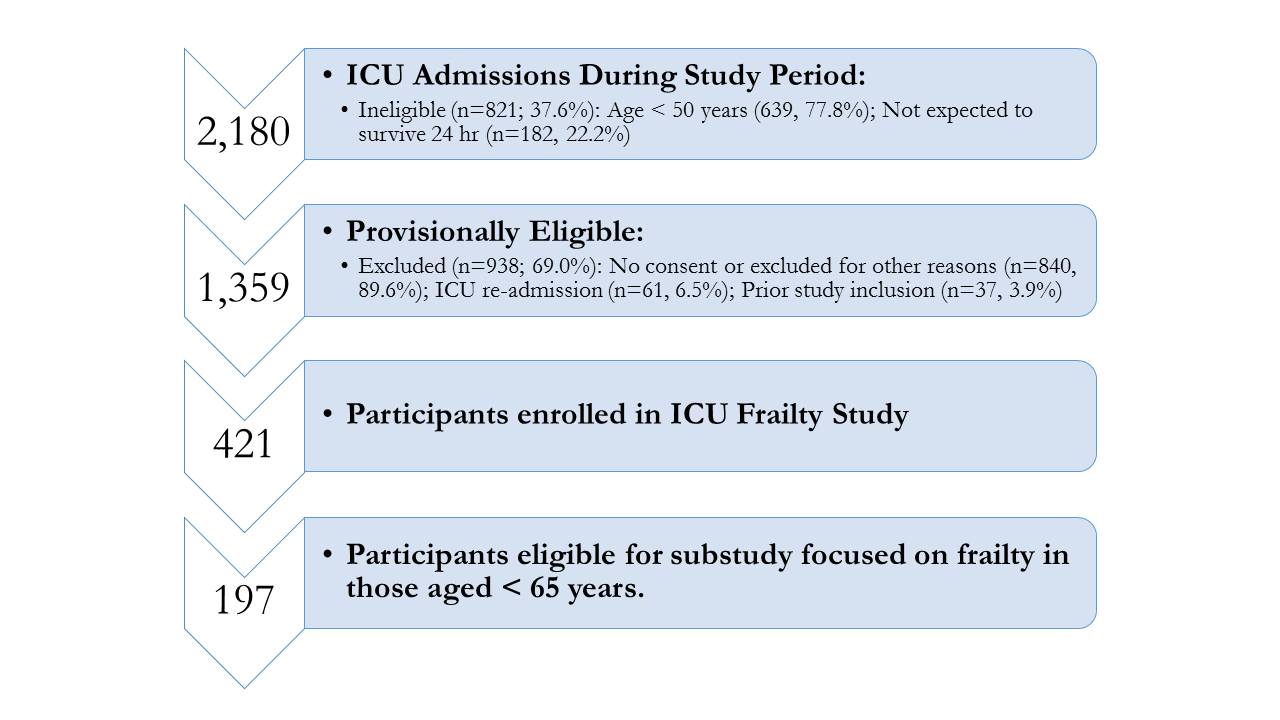

Supplement: Additional file 1: — Summary of participant flow in the study. (TIF 171 kb) [file 13054_2016_1338_MOESM1_ESM.tif]
