# Supplementary material for: A prospective multicenter cohort study of frailty in younger critically ill patients
Source: Crit Care. 2016 Jun 6;20:175. doi: 10.1186/s13054-016-1338-x (PMC4893838; doi:10.1186/s13054-016-1338-x)
Supplement: Additional file 2: — Comparison of baseline sociodemographic, clinical, and comorbidity data for patients in the full study cohort and the subgroup aged <65 years old admitted to the ICU, stratified by frailty status. (DOCX 22 kb) [file 13054_2016_1338_MOESM2_ESM.docx]

**Additional file 2:** Comparison of baseline socio-demographic, clinical and comorbidity data for patients the full study cohort and the subgroup aged <65 years old admitted to ICU stratified by frailty status.

| **Variable** | **Full Study**  **Cohort**  **(n=421)** | **Subgroup**  **(Age < 65 years)**  **(n=197)** |
| --- | --- | --- |
| Age, yr, (mean ± SD) | 67.1 (9.9) | 58.5 (4.1) |
| Sex, female (n, %) | 163 (38.7) | 72 (36.5) |
| Widowed (n, %) | 53 (12.6) | 11 (5.6) |
| Education (n, %) |  |  |
| *Less than secondary school* | 93 (22.5) | 31 (15.74) |
| *Secondary school* | 169 (40.8) | 81 (41.12) |
| *Higher level degree* | 152 (36.1) | 85 (43.15) |
| Employment status (n, %) |  |  |
| *Full time* | 72 (17.1) | 59 (29.9) |
| *Part time* | 24 (5.7) | 16 (8.1) |
| *On disability* | 65 (15.4) | 59 (29.9) |
| Pre-hospital residence |  |  |
| *At home (independent)* | 302 (71.7) | 146 (74.1) |
| *At home (with help)/other* | 99 (23.5) | 44 (22.3) |
| *Other* | 20 (4.8) | 7 (3.6) |
| CSHA functional scale score |  |  |
| *Eating (independent)* | 411 (97.6) | 193 (97.9) |
| *Dressing (independent)* | 384 (91.2) | 188 (95.4) |
| *Personal care (independent)* | 381 (91.2) | 187 (94.9) |
| *Walking (independent)* | 336 (79.8) | 169 (85.8) |
| *Getting out of bed (independent)* | 377 (89.5) | 181 (91.9) |
| *Taking bath (independent)* | 353 (83.8) | 177 (89.8) |
| *Using toilet (independent)* | 394 (93.6) | 189 (95.9) |
| *Using telephone (independent)* | 404 (96.0) | 194 (98.5) |
| *Going shopping (independent)* | 316 (75.1) | 158 (80.2) |
| *Preparing own meals (independent)* | 346 (82.2) | 167 (84.8) |
| *Doing housework (independent)* | 313 (74.3) | 158 (80.2) |
| *Taking medicine (independent)* | 360 (85.5) | 173 (87.8) |
| *Managing own finances (independent)* | 375 (89.1) | 184 (93.4) |
| Elixhauser comorbidity score, (mean±SD) | 7.7 (7.9) | 7.2 (8.1) |
| *Hypertension* | 252 (59.9) | 99 (50.3) |
| *Heart Failure* | 67 (15.9) | 19 (9.6) |
| *Diabetes mellitus* | 118 (28.0) | 49 (24.9) |
| *Chronic kidney disease* | 79 (18.8) | 36 (18.3) |
| *Rheumatoid/connective tissue disease* | 78 (18.5) | 33 (16.8) |
| *Any cancer* | 34 (8.1) | 21 (10.7) |
| *Alcohol/drug abuse* | 92 (21.9) | 66 (33.5) |
| *Psychosis* | 9 (2.1) | 7 (3.6) |
| *Depression* | 98 (23.3) | 56 (28.4) |
| Prescription medications, no. (mean±SD) | 6.6 (5.0) | 6.1 (5.2) |
| Prior hospitalization (1-year) | 176 (42.1) | 80 (40.6) |
