# Supplementary material for: A prospective multicenter cohort study of frailty in younger critically ill patients
Source: Crit Care. 2016 Jun 6;20:175. doi: 10.1186/s13054-016-1338-x (PMC4893838; doi:10.1186/s13054-016-1338-x)
Supplement: Additional file 3: — Multivariable Cox proportional hazards model HR for death in the complete study cohort (n = 421). (DOCX 18 kb) [file 13054_2016_1338_MOESM3_ESM.docx]

**Additional file 3.** Multivariable Cox PH model for hazard ratio (HR) for death in the study population (n=421)

| **Variable** | **Adjusted HR**  **(95% CI)** | **p** |
| --- | --- | --- |
| Frailty |  |  |
| No | 1 |  |
| Yes | 2.03 (1.30, 3.16) | 0.002 |
| Age < 65 years |  |  |
| No | 1 |  |
| Yes | 0.68 (0.42, 1.09) | 0.112 |
| Interaction term: Frailty ˟ Age < 65 years | 0.84 (0.42, 1.71) | 0.638 |
| Sex |  |  |
| Male | 1 |  |
| Female | 0.99 (0.70, 1.41) | 0.968 |
| Elixhauser comorbidity score | 1.03 (1.01, 1.06) | 0.002 |
| APACHE II score | 1.04 (1.01, 1.06) | 0.003 |
| Diagnostic classification |  |  |
| Sepsis | 1 |  |
| Cardiovascular | 1.19 (0.67, 2.11) | 0.560 |
| Respiratory | 0.87 (0.53, 1.43) | 0.582 |
| Gastro/ liver | 0.82 (0.43, 1.56) | 0.554 |
| Other | 0.89 (0.50, 1.58) | 0.685 |
| Hospital type |  |  |
| Tertiary care/academic | 1 |  |
| Community | 1.03 (0.71, 1.50) | 0.863 |
